# Comparative evaluation of fresh and lyophilized Nile tilapia fish skin for enhancing wound healing in a donkey model

**DOI:** 10.1007/s11259-025-10821-w

**Published:** 2025-07-23

**Authors:** Kamal H. Hussein, Mahmoud Soliman, Mahmoud Abd-Elkareem, Ahmed Abdelrahiem Sadek

**Affiliations:** 1 Department of Surgery, Anesthesiology and Radiology, Faculty of Veterinary Medicine, Assiut University, Assiut, 71516 Egypt; 2 Tissue Culture and Stem Cells Unit, Molecular Biology Researches & Studies Institute, Assiut University, Assiut, 71526 Egypt; 3 Department of Pathology and Clinical Pathology, Faculty of Veterinary Medicine, Assiut University, 71516 Assiut, Egypt; 4 Department of Immunology, University of Texas Southwestern Medical Center, Dallas, TX 75390 USA; 5 Department of Cell and Tissues, Faculty of Veterinary Medicine, Assiut University, Assiut, 71526 Egypt

**Keywords:** Wound healing, Lyophilized fish skin, Skin regeneration, Donkeys

## Abstract

Recent advancements in wound care have explored the use of biological dressings, including fish skin, due to its rich collagen content and bioactive components that promote healing. This study aimed to evaluate the effectiveness of fresh and lyophilized Nile tilapia skin in enhancing full thickness skin wound healing in donkeys. Nile tilapia skin was collected, thoroughly washed, lyophilized, and sterilized. Five female donkeys were used in the study, each receiving three full-thickness skin wounds (2 cm x 2 cm) on each side of the back after aseptic preparation and local anesthesia, resulting in a total of six wounds per animal. The wounds were assigned to three groups: control (treated with saline), fresh fish skin, and lyophilized fish skin. Macroscopic wound assessment was performed and skin samples were collected on days 14 and 28 for histological examination using hematoxylin and eosin (H&E) and Crossman’s trichrome staining. Results revealed that treatment with lyophilized fish skin significantly accelerated wound contraction and epithelialization compared to the control and fresh fish skin-treated groups. On day 14, wound contraction rates were 43.57% ± 0.87 for lyophilized fish skin, 41.32% ± 0.26 for fresh fish skin, and 32.48% ± 0.39 for the control. By day 28, contraction rates increased to 74.37% ± 0.77, 66.92% ± 0.31, and 56.88% ± 0.73, respectively. Histological analysis showed enhanced collagen deposition and angiogenesis in the lyophilized fish skin group. In conclusion, lyophilized Nile tilapia skin is a promising and cost-effective biomaterial for enhancing wound healing, offering a practical solution for field veterinarians in low-resource settings.

**Supplementary Information:**

The online version contains supplementary material available at 10.1007/s11259-025-10821-w.

## Background

The skin serves as the largest organ of the body, acting as a vital barrier that protects against environmental insults, prevents dehydration, and plays an essential role in thermoregulation and immune defense. Maintaining the integrity of the skin is crucial for overall health and well-being in both humans and animals (Nguyen and Soulika 2019; Biggs et al. 2020). In veterinary medicine, wounds pose significant challenges, particularly in equines, where the high prevalence of traumatic injuries often stems from their environment, behavior, and anatomical predisposition. A recent retrospective study conducted at an equine clinic in Cairo from 2019 to 2022 revealed that wounds were the leading cause of hospitalization, accounting for 28.9% of cases among equids, including donkeys (Benedetti et al. 2024). Another study focusing on working donkeys in Egyptian brick kilns reported an alarmingly high wound prevalence of 80% (Farhat et al. 2020). These injuries were primarily attributed to factors such as poorly fitted harnesses, overloading, and mistreatment.

These wounds can result from various causes, including sharp objects, accidents, surgical procedures, or prolonged pressure on certain areas. If left untreated or inadequately managed, such wounds may lead to infections, chronic inflammation, and delayed healing, ultimately impacting the animal’s productivity and welfare (Lux 2022). Over the years, several therapeutic strategies have been employed to enhance wound healing in veterinary practice. Various treatments, including biological dressings, synthetic scaffolds, and pharmacological agents, have been explored to accelerate tissue repair and reduce complications. However, many of these approaches have limitations, such as high costs, inconsistent efficacy, the need for specialized preparation, or logistical challenges in field settings (Cheng et al. 2018; Hussein et al. 2019, 2025b; Pereira et al. 2019; Murphy et al. 2020; Tashkandi 2021; Sebbagh et al. 2023; Rahman et al. 2024; Motiea et al. 2025).

Recently, fish skin has emerged as a novel biomaterial for wound healing due to its unique composition and structural properties (Ibrahim et al. 2020). Rich in omega-3 fatty acids, collagen, and other bioactive molecules, fish skin provides an ideal matrix for supporting cell migration, angiogenesis, and tissue regeneration (Esmaeili et al. 2023; Zou et al. 2024). It has been successfully applied in human medicine for burns and chronic wounds, and its potential in veterinary applications is promising (Kirsner et al. 2020). However, fresh fish skin poses practical challenges, such as the risk of contamination and short shelf life (Borger et al. 2024; Ibrahim et al. 2024; Mukherjee et al. 2024). These disadvantages underline the need for alternative approaches to harness the benefits of fish skin.

In this study, we aim to investigate the effect of fresh and lyophilized fish skin on wound healing in a donkey model. Lyophilization, or freeze-drying, preserves the bioactive components of fish skin while eliminating water content, thereby enhancing its stability, shelf life, and ease of use (Ibrahim et al. 2024). By exploring this approach, we hope to provide a novel, cost-effective, and practical solution for managing skin wounds in equines, addressing the limitations of existing therapies. We hypothesized that both fresh and lyophilized Nile tilapia skin would enhance wound healing in donkeys, with the lyophilized form offering comparable efficacy while providing better practicality and stability for use in field conditions.

## Materials and methods

The experimental protocol adhered to Egyptian regulations and the OIE animal welfare guidelines for the ethical care and use of animals in research and education. The study was approved by the Research Ethics Committee of the Faculty of Veterinary Medicine, Assiut University, Assiut, Egypt (Approval No. 06/2025/0306).

### Fish skin harvest

**Fig. 1:**
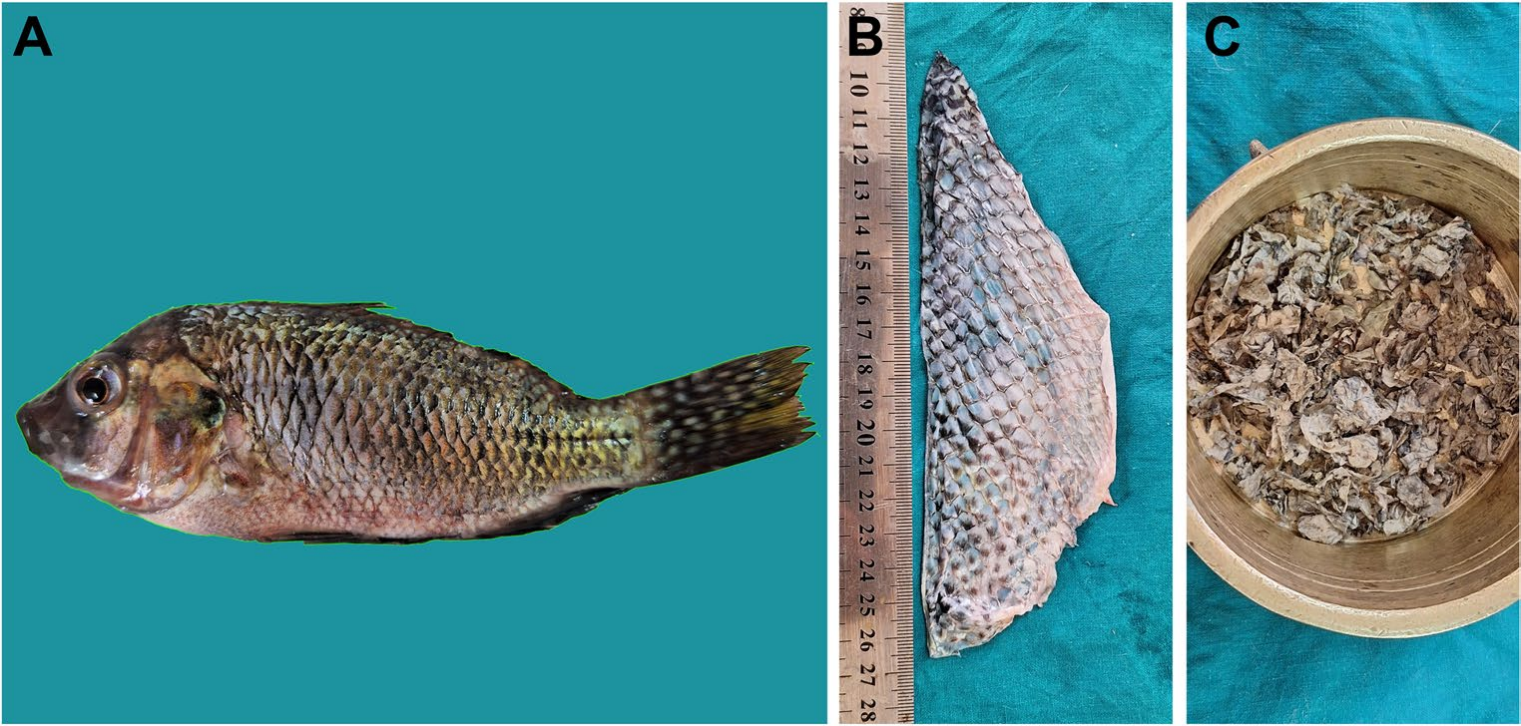
Preparation of fish skin. **A** Whole fish before skin harvesting. **B** Excised fish skin. **C** Fish skin after lyophilization

The Nile tilapia (*Oreochromis niloticus*) has been used as a source of fish skin. Fresh tilapias (300 ± 50 g) were acquired from the tanks of The Aquatic Animal Medicine Unit, Faculty of Veterinary Medicine, Assiut University. Fish scales were removed followed by dissection of fresh skin from the underlying tissues on each side of the fish as shown in Fig. 1. These collected fresh skin subjected to sectioning into rectangular pieces of approximately 3 × 3 cm in size then washed thoroughly in sterile saline containing antibiotic (Vetrocin; El-Nasr Pharmaceutical Chemicals Company, Egypt). Finally, fresh fish skin specimens were sterilized through direct exposure to ultraviolet (UV) rays for 30 min.

### Preparation of the lyophilized fish skin

The obtained sterile fish skin pieces were placed into sterile specimen cups and preserved at– 80 °C for freezing. The frozen fish skin strips were fragmented and grinded into small pieces that then were subjected to freeze dryer lyophilization (VirTis, model #6KBTES-55, Albany, NY, USA). The lyophilized fish skin fragments underwent UV sterilization and then kept in sealed cups for further application.

### Wound creation

Five apparently healthy adult female donkeys, aged 18–24 months, were acclimatized for 10 days in a controlled environment at the Veterinary Teaching Hospital, Faculty of Veterinary Medicine, Assiut University. For skin wound induction, each donkey was tranquilized by intravenous administration of xylazine hydrochloride at a dose of 1.1 mg/kg body weight.

The dorsal region of each animal was clipped and aseptically prepared using povidone-iodine solution. Following local anesthesia with 2% lidocaine, three full-thickness skin wounds, each measuring 2 × 2 cm, were carried out on each side of the aseptically prepared back, with a 3 cm distance between each wound. Wounds (*n* = 30) were assigned according to the treatment protocol into three groups (*n* = 10 for each group); control, lyophilized fish skin, and fresh fish skin-treated groups. The cranial wound was served as a control and irrigated with 0.9% normal saline, the middle wound treated with lyophilized fish skin, and the caudal wound was treated with fresh fish skin. The fresh fish skin was carefully cut to match the size and shape of each wound, then applied directly onto the wound bed without the use of adhesive agents. The treated area was subsequently covered with a sterile bandage to secure the fish skin in place. In case of lyophilized fish skin, an appropriate amount (around 500 mg) was weighed and applied to cover the wound area. The dressing was left in situ and allowed to remain until it naturally degraded or detached during the healing process. Postoperative pain management was provided using flunixin meglumine 1.1 mg/kg IV once daily for 5 days (Flunixin, Norbrook, UK). Animals were monitored daily for signs of pain, discomfort, or infection at the wound sites, and appropriate care was provided throughout the study duration.

Wounds were photographed on days 14 and 28 to evaluate the healing process. Additionally, tissue samples, including part of the surrounding normal skin, were collected for further histopathological and histomorphological analysis. Each wound was biopsied only once, allowing for more accurate evaluation of wound contraction and area over time. Skin excision was performed using a sterile surgical scalpel and scissors to create uniform full-thickness wound.

### Macroscopic analysis of wounds

The animals were followed for 28 days, and the wound was photographed at a fixed distance with a standardized ruler included. Percentage of wound contraction and wound epithelization was measured using ImageJ software, with calibration applied to each image. Wound perimeters were traced and area was caluclated independently by two observers. All measuremetns wered calculated using the following formulas and as described before (Sardari et al. 2009; Ibrahim et al. 2020):

1. *Wound size* % *on day* (*x*) *compared to day* (0) = *wound size at day* (*x*) *mm*^2^ / *wound size at day* (0) *mm*^2^ × 100
2. *Wound contraction* % = 100 - *wound size* % *at day* (*x*)
3. *Epithelialization *% = *size of epithelialization area at day* (*x*) *mm*^2^ / *size of the wound at day* (*x*) *mm*^2^ × 100

### Histopathological examination

Skin wound tissue samples, including the wound area and the surrounding healthy skin, were excised from each animal at days 14 and 28 post-wounding. The excised tissue samples were immediately fixed in 10% neutral buffered formalin. The fixed tissues were then processed for hematoxylin and eosin staining. A separate set of tissue sections was stained with Crossman’s trichrome to visualize collagen fibers and distinguish collagen in green from other tissue components. The stained slides were examined under a light microscope for histological evaluation and quantitative analysis.

### Histomorphometric analysis

Quantitative analysis of the stained sections was conducted to evaluate wound healing parameters. Microscopic fields were selected systematically from the central superficial area of each wound section, avoiding the wound margins and deep dermis to ensure homogeneity in sampling (Diegelmann and Evans 2004). All images were coded before analysis to ensure that evaluators were blinded to treatment group allocation, minimizing subjective bias. ImageJ analysis software was used to measure: the thickness of new growing epidermis (was calculated as the mean of five measurements equally spanning its length), the epithelial gap (by measuring the distance between the growing epidermis at the wound edges), inflammatory cells count, and number and average size of the newly formed blood vessels at the wound site (Wise et al. 2018; Tan et al. 2019; Ibrahim et al. 2020). Granulation tissue thickness was measured by a semiquantitative method using a scale of 1–3: 1 = mild (thin granulation tissue around wound edges); 2 = moderate (moderate granulation tissue around wound edges and in 30–50% of the wound); 3 = complete (thick granulation tissue around wound edges and in > 50% of the wound) (Tan et al. 2019; Ibrahim et al. 2020). Additionally, collagen density was quantified using ImageJ software by converting the images to RGB format, selecting the green channel representing collagen, and using a consistent threshold based on the control slides to binarize collagen-positive areas and applied uniformly across all samples to ensure reproducibility. The area fraction (%) of collagen was calculated as the percentage of the positively stained area relative to the total tissue area (Cheng et al. 2018). Statistical results were derived from histological assessments conducted on *n* = 5 wounds per group per time point (Sadiq et al. 2018; Soliman et al. 2021).

### Statistical analysis

Data was analyzed by one-way analysis of variance (ANOVA) followed by Tukey’s post hoc test using GraphPad Prism software version 8 (GraphPad Software Inc., La Jolla, CA, USA) and was applied separately for each time point, and that no across-time statistical comparisons were conducted. Results are presented as mean ± standard deviation, with statistical significance set at *p* < 0.05.

## Results

### Macroscopic findings

**Fig. 2:**
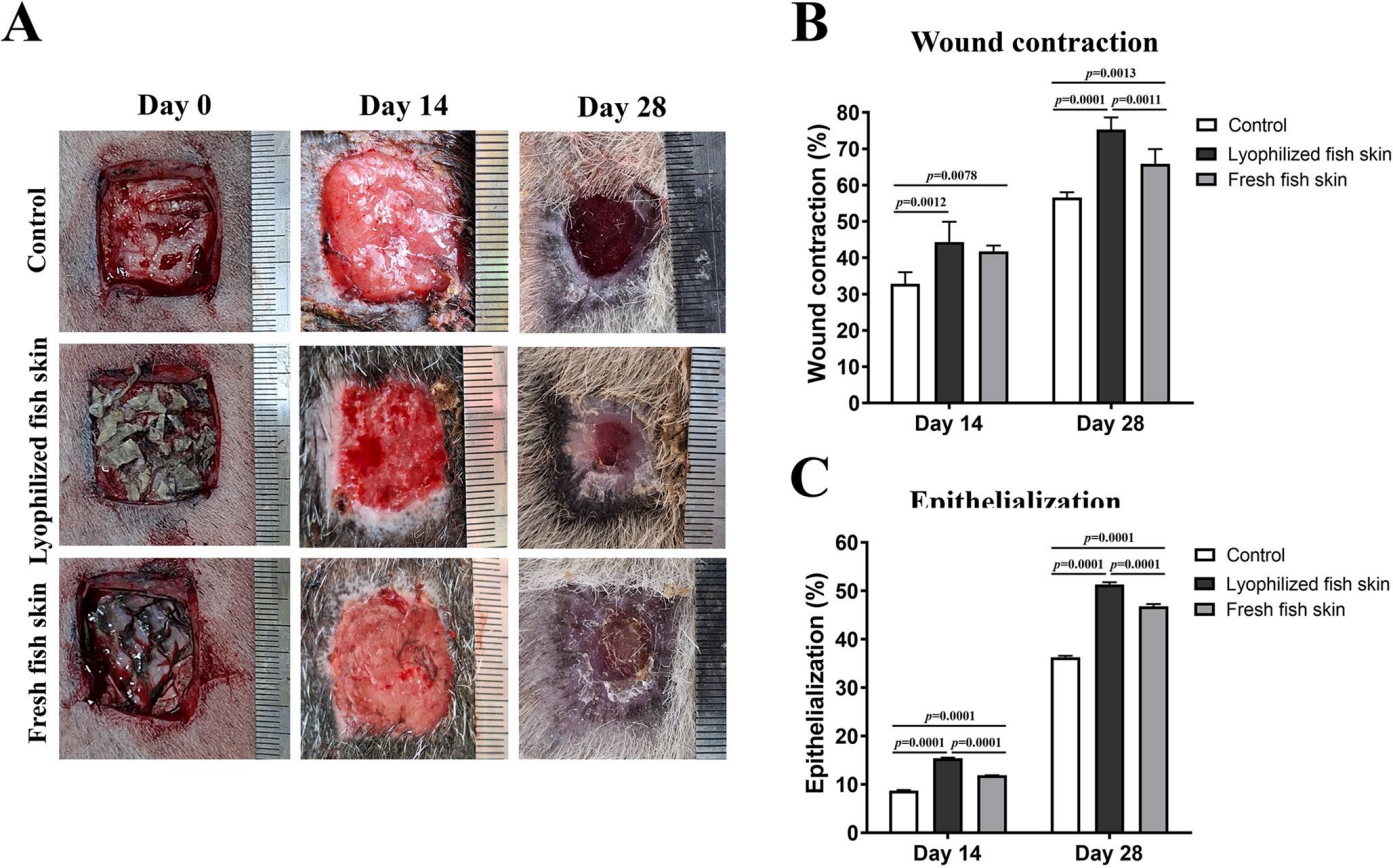
Gross assessment of the skin wound in donkeys dressed with or without lyophilized or fresh fish skin. **A** Representative images of skin wounds from the control group or fish skin groups on days 0, 14 and 28. **B** Percentage of wound closure (**B**) and epithelialization (**C**) were quantified using ImageJ and compared to the control group

The application of lyophilized and fresh fish skins significantly accelerated wound healing compared to the control group. On day 14, wounds treated with lyophilized fish skin showed a 44.38% ± 5.56 (*p* = 0.0012) wound contraction rate, while those treated with fresh fish skin achieved a 41.77% ± 1.57 (*p* = 0.0078) wound contraction rate, compared to 32.87% ± 3.14 in the control group (Fig. 2A and B) (Supplementary Fig. 1). By day 28, wound contraction rates increased to 75.36% ± 3.30 (*p* = 0.0001) for the lyophilized fish skin treated group and 65.90% ± 4.01 (*p* = 0.0013) for the fresh fish skin treated group, compared to 56.63% ± 1.41 in the control group (Fig. 2A and B) (Supplementary Fig. 1). Furthermore, wounds treated with lyophilized and fresh fish skin showed an increase in the percentage of epithelialization on days 14 and 28 (15.40% ± 0.15 and 51.28% ± 0.51 for lyophilized fish skin (*p* = 0.0001); 11.89% ± 0.12 and 46.82% ± 0.47 for fresh fish skin (*p* = 0.0001), respectively) compared to the control wounds (8.75% ± 0.09 and 36.24% ± 0.36, respectively) (Fig. 2C). In comparison between both the fresh and lyophilized fish skin, the lyophilized fish skin achieved the highest wound contraction and epithelialization rates than the fresh fish skin (Fig. 2B and C).

### Histopathological findings

**Fig. 3:**
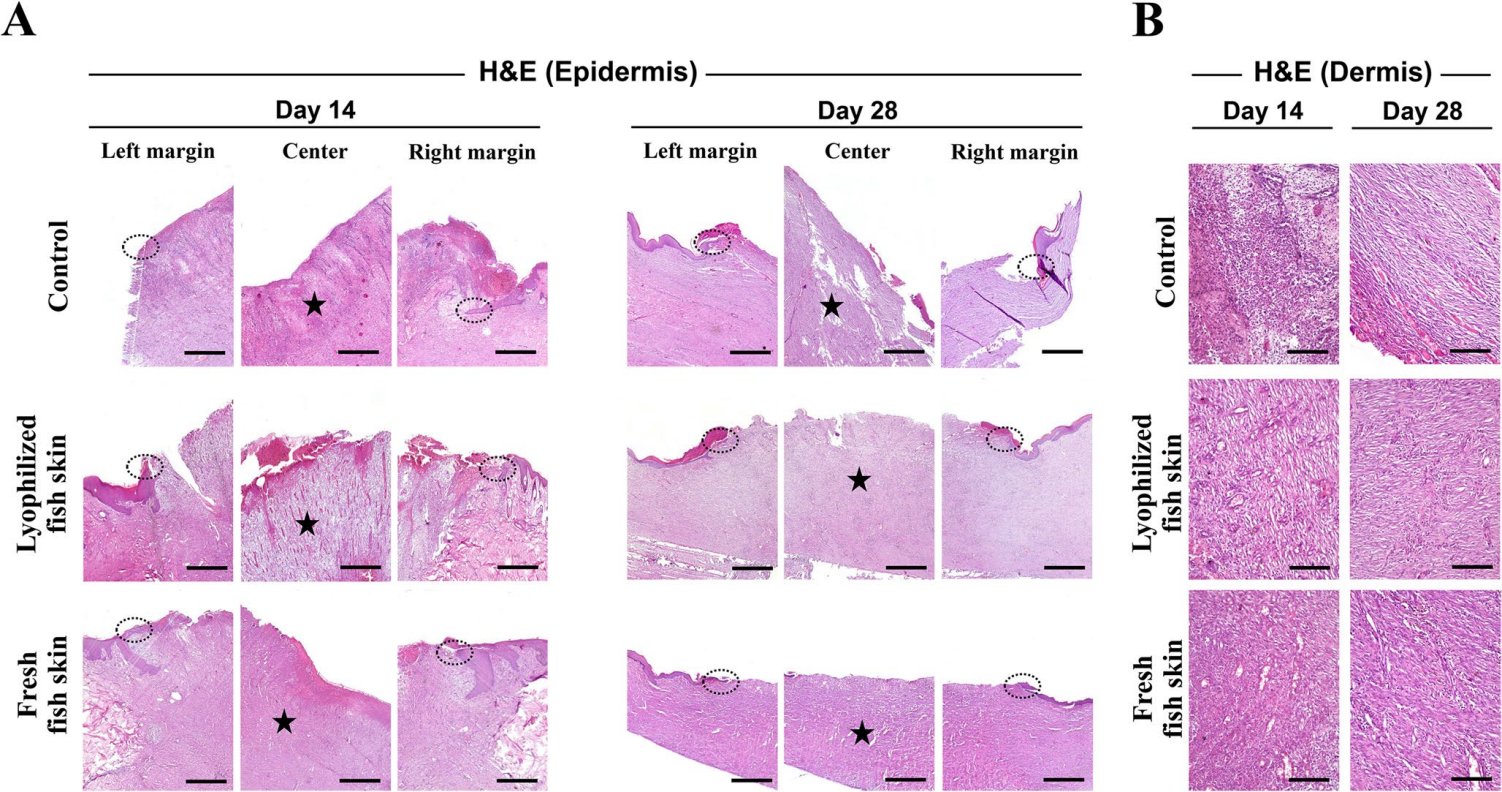
Histopathological evaluation of the skin wound in donkeys dressed with or without lyophilized or fresh fish skin. Representative histopathological images from the wound sections in the control, lyophilized fish skin, and fresh fish skin groups on days 14, and 28 post-wound induction showing the epidermis (**A**) with delayed re-epithelialization and inflammatory cells infiltration in the control group and improved re-epithelialization and less inflammatory cells infiltration in the fish skin groups, and dermis (**B**) with inflammatory cells infltrates and visible blood vessel fromation. Dotted circles indicate the growing epithelial ends. Stars indicate inflammatory cells infiltrate. Tissue sections were stained Hematoxylin and Eosin. Scale bars: (**A**) = 100 μm and (**B**) = 50 μm

**Fig. 4:**
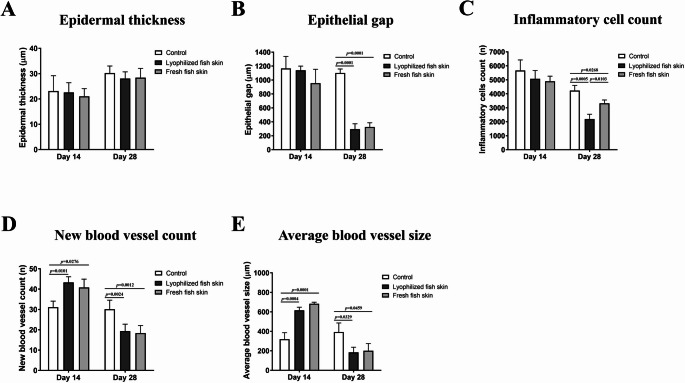
Histomorphometrical assessment of the skin wound healing. **A** Epidermal thickness, **B** epithelial gap, **C** inflammatory cell infiltration, **D** number of new blood vessels, and **E** size of newly formed blood vessels in wound sections from the control, lyophilized fish skin, and fresh fish skin groups were measured using ImageJ on days 14, and 28 post-wound induction

Histopathological analysis of the wounds at days 14 and 28 post-wounding was performed to assess and compare the key parameters of wound healing between the control and fresh and lyophilized fish skin groups. Wounds treated with either lyophilized or fresh fish skin showed regenerated epidermis as the growing epithelial cells were recognized based on their elongated, basophilic nuclei, palisading arrangement, and location at the wound edges, forming a continuous epithelial tongue migrating over the granulation tissue (Fig. 3A). There was no significant difference in the epidermal thickness among the control, lyophilized fish skin, and fresh fish skin groups (Fig. 4A). The epithelial gap, as indicator of wound closure and analyzed by measuring the distance between the newly formed epidermal tips on both wound edges, was not significant different at day 14 post-wounding (1139.60 μm ± 60.67 and 953.71 μm ± 201.17, respectively) compared to control wounds (1166.77 μm ± 172.27) (Figs. 3A and 4B). However, at day 28 post-wounding, lyophilized or fresh fish skin significantly (294.27 μm ± 77.77 and 326.02 μm ± 60.27, respectively) (*p* = 0.0001) enhanced epidermal gap closure compared to control wounds (1101.07 μm ± 57.77) (Figs. 3A and 4B).

The recruitment of inflammatory cells, consisted primarily of neutrophils and macrophages, in the wound site was induced in the early stage of healing (day 14 post-wounding) with no significant difference between the lyophilized and fresh fish skin and control groups (Figs. 3B and 4C). At a later time-point (day 28 post-wounding), the number of inflammatory cells decreased in the lyophilized (*p* = 0.0005) and fresh (*p* = 0. 0268) fish skin groups but remained high in the control wounds (Figs. 3B and 4C). The lyophilized fish skin-treated group had a lower number of inflammatory cells than the fresh fish skin-treated group at day 28 post-wounding (*p* = 0.0103) (Fig. 4C).

Next, we investigated angiogenesis, a fundamental process in the early stages of wound healing, at the wound site. There was a significant increase in both the number and size of the new blood vessels at the wound site in the lyophilized and fresh fish skin groups (number of new blood vessels *p* = 0.0101 and *p* = 0.0276, respectively; and size of new blood vessels *p* = 0.0004 and *p* = 0.0001, respectively) compared to the control group on day 14 post-wounding (Figs. 3B and 4D and E). However, on day 28 post-wounding, the lyophilized and fresh fish skin groups had fewer (*p* = 0.0024 and *p* = 0.0012, respectively) and smaller blood vessels (*p* = 0.0329 and *p* = 0.0459, respectively) than the control group (Figs. 3B and 4D and E). Both lyophilized and fresh fish skin showed similar effects on angiogenesis, with no statistically significant differences in blood vessel number or size at either time point (Fig. 4D and E).

### Assessment of the granulation tissue

**Fig. 5:**
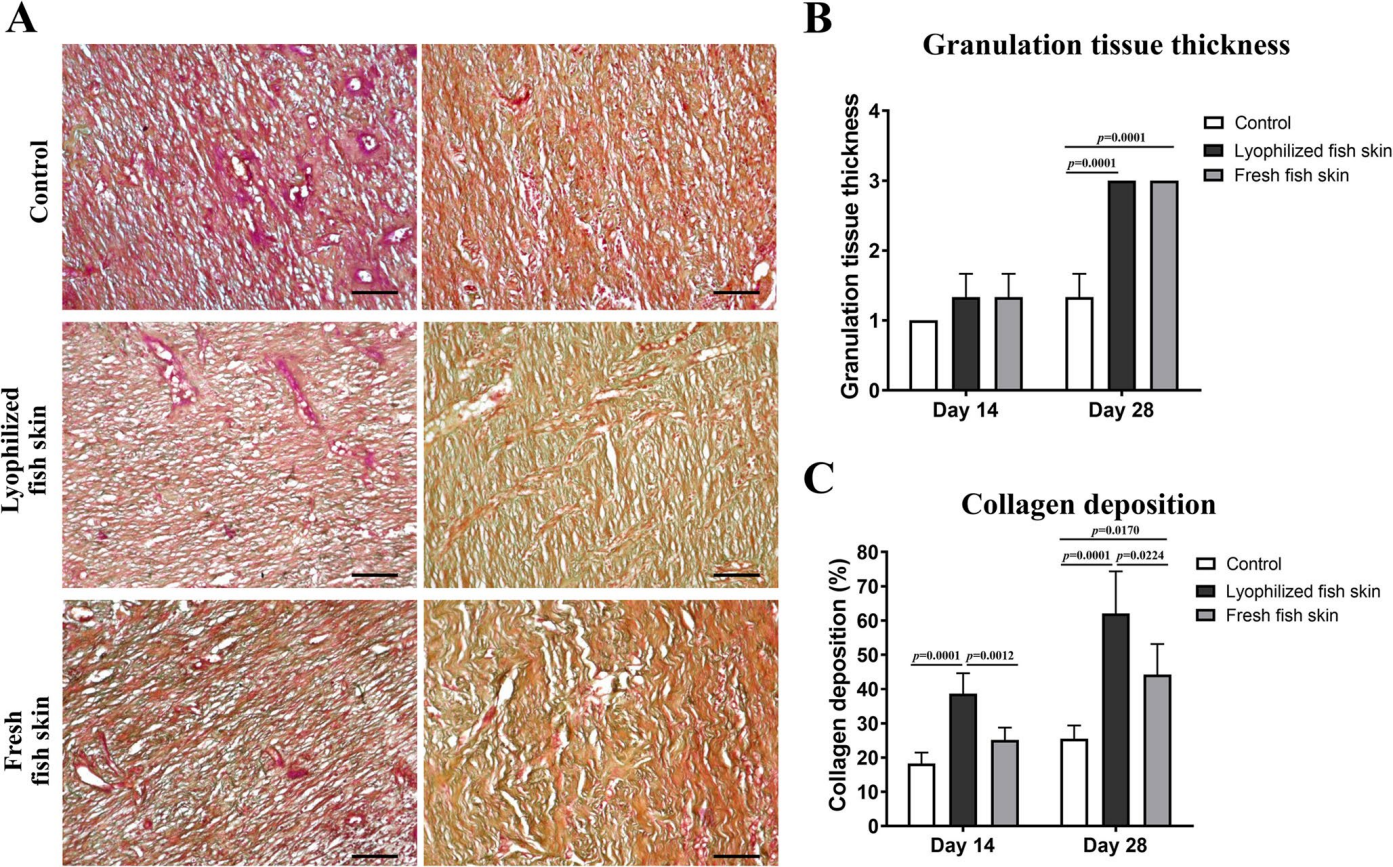
Evaluation of the granulation tissue in the wound area. **A** Representative images of wound sections from the control, lyophilized fish skin, and fresh fish skin groups were stained with Crossman’s Trichrome on days 14, and 28 post-wound induction. Collagen fibers appear green and fiber organization appeared well-aligned in the lyophilized and fresh fish skin groups, not in the control group. Scale bars = 50 μm. **B** Granulation tissue thickness was evaluated on a 1–3 scale. **C** Collagen deposition was quantified using ImageJ and expressed as a percentage of the total pixel count and normalized to the control group

Crossman’s trichrome staining of the wound sections demonstrated fibroblast proliferation and granulation tissue formation in both fresh and lyophilized fish skin groups with well-organized fibers and increased collagen deposition, while the control group showed disorganized fibers and less collagen deposition (Fig. 5A). On day 14 post-wound induction, no significant difference was observed in the thickness of the granulation tissue between the control, lyophilized fish skin, and fresh fish skin groups (Fig. 5B), with significant increase in the collagen deposition in the lyophilized fish skin group (*p* = 0.0001), not in the fresh fish skin group (*p* = 0.0734), compared to the control (Fig. 5C). However, by day 28, both fresh and lyophilized fish skin groups exhibited increased in the thickness of the granulation tissue (*p* = 0.0001) and collagen deposition (*p* = 0.0001 and *p* = 0.0170, respectively) compared to the control (Fig. 5B and C). Notably, the lyophilized fish skin group showed slightly higher collagen intensity than the fresh fish skin group (*p* = 0.0224), suggesting enhanced extracellular matrix remodeling (Fig. 5C). These results indicate that while both lyophilized and fresh fish skin promote wound healing, lyophilized fish skin may have a more pronounced effect on collagen synthesis.

## Discussion

This study aimed to evaluate the efficacy of fresh and lyophilized fish skin in promoting wound healing in donkeys. Our findings demonstrated that both treatments significantly enhanced wound contraction and epithelialization compared to control wounds treated with saline. Notably, lyophilized fish skin exhibited superior outcomes, suggesting its potential as a practical and effective wound care solution in veterinary medicine.

Fish skin has garnered attention as a promising biomaterial for wound healing due to its unique composition. Rich in type I collagen, omega-3 polyunsaturated fatty acids (PUFAs), and essential peptides, fish skin provides a conducive environment for tissue regeneration. Type I collagen serves as a structural scaffold, facilitating cell migration and proliferation, which are critical for tissue repair. Omega-3 PUFAs, such as eicosapentaenoic acid (EPA) and docosahexaenoic acid (DHA), possess anti-inflammatory properties that modulate the wound environment, reducing excessive inflammation and promoting healing (Calder 2010). Additionally, bioactive peptides derived from fish skin have been shown to exhibit antimicrobial and immunomodulatory effects, further supporting the wound healing process (Esmaeili et al. 2023; Zou et al. 2024). Inflammation is essential for wound healing to fight infection and clear debris. It then shifts from a proinflammatory to a healing phase, allowing tissue repair. However, persistent inflammation can lead to excessive scarring or chronic wounds (Wynn and Vannella 2016; Eming et al. 2017). Our findings indicate that both lyophilized and fresh fish skin hastened the resolution of wound inflammation, with the lyophilized fish skin exhibiting a lower inflammatory reaction on day 28. In the treated groups, the observed reduction in vascular density by day 28 may suggest an accelerated transition to the remodeling phase. Enhanced therapeutic effects, such as improved extracellular matrix deposition, reduced inflammation, and early re-epithelialization, could contribute to the resolution of the angiogenic stimulus, leading to earlier pruning of newly formed vessels and stabilization of the vasculature (Li et al. 2003; Werner and Grose 2003). However, the mechanism underlying this reduced inflammation requires further investigation.

The components of fish skin play pivotal roles in angiogenesis and granulation tissue formation—key processes in wound healing. Type I collagen provides a matrix that supports endothelial cell adhesion and migration, essential steps in new blood vessel formation (Zimba et al. 2024). Fibroblast growth factors (FGFs) present in fish skin stimulate the proliferation of fibroblasts and endothelial cells, enhancing granulation tissue development and neovascularization. The presence of omega-3 PUFAs further augments angiogenesis by modulating inflammatory responses and supporting endothelial function (Biazar et al. 2022; Zhao et al. 2024). Consistently, in wounds treated with both lyophilized and fresh fish skin, both the number and size of new blood vessels increased, which may be attributed to the high content of omega-3 PUFAs and collagen in the fish skin. Omega-3 PUFAs have been shown to enhance angiogenesis by promoting endothelial cell proliferation and formation via modulation of the VEGF signaling pathway. Similarly, fish skin collagen provides a biocompatible scaffold rich in type I collagen, which supports endothelial cell adhesion and neovascularization during wound repair (Zhuang et al. 2013; Biazar et al. 2022; Zhao et al. 2024). These features suggest that wound healing may be regulated in part by blood vessel formation.

In addition, both lyophilized and fresh fish skin promoted granulation tissue thickness and increased collagen deposition in the wound tissue, with the lyophilized fish skin exhibiting a noticeable rise in collagen density. This explained the decrease in wound size in the fish dressed group more than the control group, with the lyophilized fish skin-dressed wound exhibiting a smaller wound size.

The granulation tissue formation with high degree of collagen deposition and angiogenesis in the healed tissue are considered as an indicator for the wound tissue maturity. Collagen provides structural support, and angiogenesis ensures an adequate supply of oxygen and nutrients to the healing area (Rodrigues et al. 2019). Furthermore, the collagen matrix in fish skin acts as a scaffold for re-epithelialization of the wound, as well as the mild inflammation and the granulation tissue formation in the wound tissue caused by the fish skin promote this process. This was demonstrated in the present study, where both lyophilized and fresh fish skin increased epithelialization rate, with the lyophilized fish skin showing the highest epithelialization rate.

While fresh fish skin has demonstrated efficacy in wound healing, its practical application faces challenges. Fresh fish skin is perishable, requiring strict storage conditions to prevent microbial contamination and degradation. Maintaining a cold chain during transportation and storage can be logistically demanding and costly, particularly in regions with limited resources. These constraints limit the widespread adoption of fresh fish skin as a wound dressing material (Ibrahim et al. 2024; Mukherjee et al. 2024).

Lyophilization, or freeze-drying, offers a viable solution to these challenges by preserving fish skin’s bioactive components while enhancing its stability and shelf life. This process involves the removal of water content under low temperatures, resulting in a dry, lightweight product that is easier to store and transport without the need for refrigeration (Nowak and Jakubczyk 2020). These properties simplify handling, improve procedural efficiency, and increase ease of use, particularly in remote or resource-limited settings. The extended shelf life also supports long-term storage and broad distribution, enhancing accessibility. While lyophilization may slightly raise initial production costs, these are offset by reduced cold-chain requirements and improved logistical efficiency (Hussein et al. 2025a). Overall, lyophilized fish skin represents a scalable and practical option for wound management across diverse clinical and field environments. Importantly, studies have shown that lyophilized fish skin retains its structural integrity and biological activity, making it an effective scaffold for tissue regeneration. Moreover, lyophilization reduces the risk of disease transmission and simplifies the handling of the graft material (Ibrahim et al. 2024).

Unlike the study by Ibrahim et al. 2020; which focused on the application of fresh fish skin to metacarpal wounds, our study offers a broader and more comparative approach. We evaluated both fresh and lyophilized fish skin dressings using a full-thickness excisional wound model on the dorsal area in donkeys, a site selected for its consistent surface area and lower susceptibility to movement-induced disruption. This allowed for a more controlled and reliable assessment of healing parameters. We also investigated the practical benefits of lyophilized fish skin, such as improved handling characteristics, increased durability, and potential applicability in the field.

Our results align with previous studies that have highlighted the benefits of fish skin in wound management. For instance, research has demonstrated that fish skin grafts accelerate re-epithelialization, reduce pain, and decrease the frequency of dressing changes in burns (Garrity et al. 2023; Borger et al. 2024).

In Fortaleza, Brazil, a 3-year-old with scald burns on 18% of his body was treated with sterilized tilapia skin. After wound cleaning and resuscitation, the tilapia skin was applied along with silver sulfadiazine and gauze. By day 6, it adhered well, and by day 10, full tissue regeneration occurred. Tilapia skin removal was painless, and recovery was complication-free (Costa et al. 2019).

Garrity et al. found that fish skin promoted twice as much vascularization and antimicrobial defensin peptide expression compared to controls. Proteomic analysis confirmed the presence of antimicrobial peptides in the fish skin (Garrity et al. 2023).

Additionally, the use of fish skin in chronic wound care has been associated with improved healing outcomes and patient satisfaction. These findings suggest that fish skin could be a cost-effective therapeutic option for burn victims, enhancing vascularization and reducing bacterial infection, and underscore the therapeutic potential of fish skin-derived products in diverse clinical settings (Luze and Nischwitz 2022).

Compared to widely used commercial xenografts such as porcine small intestinal submucosa (SIS), fish skin offers several distinct advantages. Its marine origin reduces the risk of zoonotic disease transmission and eliminates concerns related to religious or cultural restrictions associated with porcine products (Fujii and Tanaka 2022). Additionally, fish skin retains a naturally rich composition of omega-3 fatty acids, which are known to modulate inflammation and promote tissue regeneration. Unlike extensively processed mammalian xenografts, fish skin requires minimal chemical treatment, preserving its native structure and bioactivity. These attributes make fish skin a promising and potentially safer alternative for wound healing, particularly in veterinary and field settings.

The limitations of this study include the small sample size may reduce the statistical power and generalizability of the findings. However, the within-subjects design, where each of the five donkeys received all three treatments across six wounds, allowed for balanced intra-animal comparisons and reduced variability. This design enhanced statistical power and justified the small sample size, which is consistent with similar studies and reflects the ethical constraints of large animal research. In this study, wounds were assigned to fixed cranial, middle, and caudal positions along the lateral thoracic area to maintain procedural consistency and reduce handling stress. However, this approach may have introduced anatomical bias, as variations in vascularity, mobility, or tension at different sites could have influenced healing outcomes. To address this, future studies should adopt randomized or rotational wound placement to minimize positional effects and improve the robustness of the findings. Additionally, the exclusive use of female donkeys is a limitation. While this selection was based on availability and aimed at reducing variability, it introduces potential gender bias. Anatomical and hormonal differences between sexes may influence wound healing outcomes, including inflammatory responses, collagen deposition, and re-epithelialization rates. Future studies should incorporate both male and female animals to ensure broader applicability and to better understand any sex-specific effects of fish skin graft treatments.

Another limitation of the current study is the use of saline as the sole control treatment. While this choice allowed for a focused comparison between fresh and lyophilized fish skin, it does not provide insights into how these materials perform relative to conventional wound therapies. Future studies should compare fish skin to established clinical treatments to better contextualize its therapeutic value in equine practice. Finally, a key limitation of this study is the absence of scar quality and functional assessments such as elasticity, pigmentation, and tensile strength. While histological analysis was performed at two time points (days 14 and 28), this does not fully capture early inflammatory responses or long-term remodeling phases such as scar maturation. Future studies should include earlier and later time points, along with biomechanical and cosmetic evaluations, to comprehensively assess healing outcomes.

In conclusion, both fresh and lyophilized fish skin significantly enhance wound healing in donkeys, with lyophilized fish skin showing superior efficacy. The practical advantages of lyophilized fish skin, including extended shelf life and ease of storage, make it a promising candidate for widespread use in veterinary wound care. Future studies should explore the long-term effects and potential applications of lyophilized fish skin in various animal species and wound types to fully elucidate its clinical benefits.

## Electronic supplementary material

Below is the link to the electronic supplementary material.

ESM 1 (PNG 853 KB)

High Resolution Image (26.4 MB)

## Data Availability

No datasets were generated or analysed during the current study.

## References

[CR1] Benedetti B, Freccero F, Barton J et al (2024) A retrospective study on the status of working equids admitted to an equine clinic in cairo: disease prevalence and associations between physical parameters and outcome. Animals 14:817 38473201 10.3390/ani14050817 PMC10930472

[CR2] Biazar E, Heidari Keshel S, Rezaei Tavirani M et al (2022) Healing effect of acellular fish skin with plasma rich in growth factor on full-thickness skin defects. Int Wound J 19:2154–2162 35441469 10.1111/iwj.13821 PMC9705163

[CR3] Biggs LC, Kim CS, Miroshnikova YA et al (2020) Mechanical forces in the skin: roles in tissue architecture, stability, and function. J Invest Dermatol 140:284–290 31326398 10.1016/j.jid.2019.06.137

[CR4] Borger A, Semmler L, Bergmann F et al (2024) Synergistic treatment of infected burn wound utilizing maggot debridement and acellular fish skin grafting—a case report. J Burn Care Res 45:1336–1340 38953562 10.1093/jbcr/irae128 PMC11379142

[CR5] Calder PC (2010) Omega-3 fatty acids and inflammatory processes. Nutrients 2:355–374 22254027 10.3390/nu2030355 PMC3257651

[CR6] Cheng K-Y, Lin Z-H, Cheng Y-P et al (2018) Wound healing in Streptozotocin-induced diabetic rats using atmospheric-pressure argon plasma jet. Sci Rep 8:12214 30111887 10.1038/s41598-018-30597-1 PMC6093903

[CR7] Costa BA, Lima Júnior EM, de Moraes Filho MO et al (2019) Use of tilapia skin as a xenograft for pediatric burn treatment: a case report. J Burn Care Res 40:714–717 31112268 10.1093/jbcr/irz085

[CR8] Diegelmann RF, Evans MC (2004) Wound healing: an overview of acute, fibrotic and delayed healing. Front Biosci 9:283–289 14766366 10.2741/1184

[CR9] Eming SA, Wynn TA, Martin P (2017) Inflammation and metabolism in tissue repair and regeneration. Sci (New York NY) 356:1026–1030 10.1126/science.aam7928 28596335

[CR10] Esmaeili A, Biazar E, Ebrahimi M et al (2023) Acellular fish skin for wound healing. Int Wound J 20:2924–2941 36924081 10.1111/iwj.14158 PMC10410342

[CR11] Farhat SF, McLean AK, Mahmoud HFF (2020) Welfare assessment and identification of the associated risk factors compromising the welfare of working donkeys (Equus asinus) in Egyptian Brick Kilns. Animals 10:1611 32917031 10.3390/ani10091611 PMC7552282

[CR12] Fujii M, Tanaka R (2022) Porcine small intestinal submucosa alters the biochemical properties of wound healing: a narrative review. Biomedicines 10:2213 36140314 10.3390/biomedicines10092213 PMC9496019

[CR13] Garrity C, Garcia-Rovetta C, Rivas I et al (2023) Tilapia fish skin treatment of third-degree skin burns in murine model. J Funct Biomater 14:512 37888177 10.3390/jfb14100512 PMC10607444

[CR14] Hussein KH, Abdelhamid HN, Zou X et al (2019) Ultrasonicated graphene oxide enhances bone and skin wound regeneration. Mat Sci Eng: C 94:484–492 10.1016/j.msec.2018.09.051 30423733

[CR15] Hussein KH, Motiea E, Hussein MT (2025a) Efficacy of xenogeneic fresh and lyophilized amniotic membranes on the healing of experimentally induced full-thickness skin wounds in dogs. Sci Rep 15:15605 40320419 10.1038/s41598-025-95023-9 PMC12050321

[CR16] Hussein KH, Soliman M, Sabra MS et al (2025b) Regenerative potential of graphene oxide-chitosan nanocomposite combined with fetal bovine serum on healing of full-thickness skin wound in rats. BMC Vet Res 21:324 40336048 10.1186/s12917-025-04721-z PMC12057232

[CR17] Ibrahim A, Soliman M, Kotb S et al (2020) Evaluation of fish skin as a biological dressing for metacarpal wounds in donkeys. BMC Vet Res 16:472 33272259 10.1186/s12917-020-02693-w PMC7713020

[CR18] Ibrahim A, Fahmy HM, Mahmoud GA et al (2024) New strategies for sterilization and preservation of fresh fish skin grafts. Sci Rep 14:1253 38218988 10.1038/s41598-024-51608-4 PMC10787751

[CR19] Kirsner RS, Margolis DJ, Baldursson BT et al (2020) Fish skin grafts compared to human amnion/chorion membrane allografts: a double-blind, prospective, randomized clinical trial of acute wound healing. Wound Repair Regen 28:75–80 31509319 10.1111/wrr.12761 PMC6972637

[CR20] Li J, Zhang YP, Kirsner RS (2003) Angiogenesis in wound repair: angiogenic growth factors and the extracellular matrix. Microsc Res Tech 60:107–114 12500267 10.1002/jemt.10249

[CR21] Lux CN (2022) Wound healing in animals: a review of physiology and clinical evaluation. Vet Dermatol 33:91–e27 34704298 10.1111/vde.13032

[CR22] Luze H, Nischwitz SP (2022) The use of acellular fish skin grafts in burn wound management-a systematic review. Medicina 58:912 35888631 10.3390/medicina58070912 PMC9323726

[CR23] Motiea E, Hussein MT, Hussein KH (2025) Investigating the healing potential of fresh amniotic membranes in full-thickness canine skin wounds. Assiut Vet Med J 71:73–80

[CR24] Mukherjee S, Bhattacherjee S, Keswani K et al (2024) Application of tilapia fish skin in treatment of burn patients. Biocatal Agric Biotechnol 59:103254

[CR25] Murphy SV, Skardal A, Nelson RA Jr et al (2020) Amnion membrane hydrogel and amnion membrane powder accelerate wound healing in a full thickness porcine skin wound model. Stem Cells Transl Med 9:80–92 31328435 10.1002/sctm.19-0101 PMC6954699

[CR26] Nguyen AV, Soulika AM (2019) The dynamics of the skin’s immune system. Int J Mol Sci 20:1811 31013709 10.3390/ijms20081811 PMC6515324

[CR27] Nowak D, Jakubczyk E (2020) The freeze-drying of foods-the characteristic of the process course and the effect of its parameters on the physical properties of food materials. Foods (Basel Switzerland) 9:1488 33080983 10.3390/foods9101488 PMC7603155

[CR28] Pereira R, De La Côrte FD, Brass KE et al (2019) Evaluation of three methods of platelet-rich plasma for treatment of equine distal limb skin wounds. J Equine Vet Sci 72:1–7 30929771 10.1016/j.jevs.2017.10.009

[CR29] Rahman E, Rao P, Abu-Farsakh HN et al (2024) Systematic review of platelet-rich plasma in medical and surgical specialties: quality, evaluation, evidence, and enforcement. J Clin Med 13:4571 39124838 10.3390/jcm13154571 PMC11313071

[CR30] Rodrigues M, Kosaric N, Bonham CA et al (2019) Wound healing: a cellular perspective. Physiol Rev 99:665–706 30475656 10.1152/physrev.00067.2017 PMC6442927

[CR31] Sadiq A, Menchetti I, Shah A et al (2018) 5-HT1A receptor function makes wound healing a happier process. Front Pharmacol 9:1406 30618734 10.3389/fphar.2018.01406 PMC6297675

[CR32] Sardari K, Kazemi H, Emami MR et al (2009) Role of collagen cross-linking on equine wound contraction and healing. Comp Clin Path 18:239–247

[CR33] Sebbagh P, Cannone A, Gremion G et al (2023) Current status of PRP manufacturing requirements & European regulatory frameworks: practical tools for the appropriate implementation of PRP therapies in musculoskeletal regenerative medicine. Bioengineering 10:292 36978683 10.3390/bioengineering10030292 PMC10044789

[CR34] Soliman M, Sadek AA, Abdelhamid HN et al (2021) Graphene oxide-cellulose nanocomposite accelerates skin wound healing. Res Vet Sci 137:262–273 34052571 10.1016/j.rvsc.2021.05.013

[CR35] Tan WS, Arulselvan P, Ng SF et al (2019) Improvement of diabetic wound healing by topical application of Vicenin-2 hydrocolloid film on Sprague Dawley rats. BMC Complement Altern Med 19:20 30654793 10.1186/s12906-018-2427-y PMC6337851

[CR36] Tashkandi H (2021) Honey in wound healing: an updated review. Open Life Sci 16:1091–1100 34708153 10.1515/biol-2021-0084 PMC8496555

[CR37] Werner S, Grose R (2003) Regulation of wound healing by growth factors and cytokines. Physiol Rev 83:835–870 12843410 10.1152/physrev.2003.83.3.835

[CR38] Wise LM, Bodaan CJ, Stuart GS et al (2018) Treatment of limb wounds of horses with orf virus il-10 and VEGF-E accelerates resolution of exuberant granulation tissue, but does not prevent its development. PLoS ONE 13:e0197223 29763436 10.1371/journal.pone.0197223 PMC5953458

[CR39] Wynn TA, Vannella KM (2016) Macrophages in tissue repair, regeneration, and fibrosis. Immunity 44:450–462 26982353 10.1016/j.immuni.2016.02.015 PMC4794754

[CR40] Zhao C, Feng M, Gluchman M et al (2024) Acellular fish skin grafts in the treatment of diabetic wounds: advantages and clinical translation. J Diabetes Res 16:e13554 10.1111/1753-0407.13554 PMC11045921 38664883

[CR41] Zhuang W, Wang G, Li L et al (2013) Omega-3 polyunsaturated fatty acids reduce vascular endothelial growth factor production and suppress endothelial wound repair. J Cardiovasc Transl Res 6:287–293 22993129 10.1007/s12265-012-9409-0

[CR42] Zimba BL, Rwiza MJ, Sauli E (2024) Utilizing tilapia fish skin biomaterial for burn wound dressing: a systematic review. Sci Afr 24:e02245

[CR43] Zou Y, Mao Z, Zhao C et al (2024) Fish skin dressing for wound regeneration: a bioactive component review of omega-3 pufas, collagen and ECM. Int J Biol Macromol 283:137831 39566781 10.1016/j.ijbiomac.2024.137831

