# Supplementary figures and images for: Comparative evaluation of fresh and lyophilized Nile tilapia fish skin for enhancing wound healing in a donkey model
Source: Vet Res Commun. 2025 Jul 23;49(5):262. doi: 10.1007/s11259-025-10821-w (PMC12287218; doi:10.1007/s11259-025-10821-w)

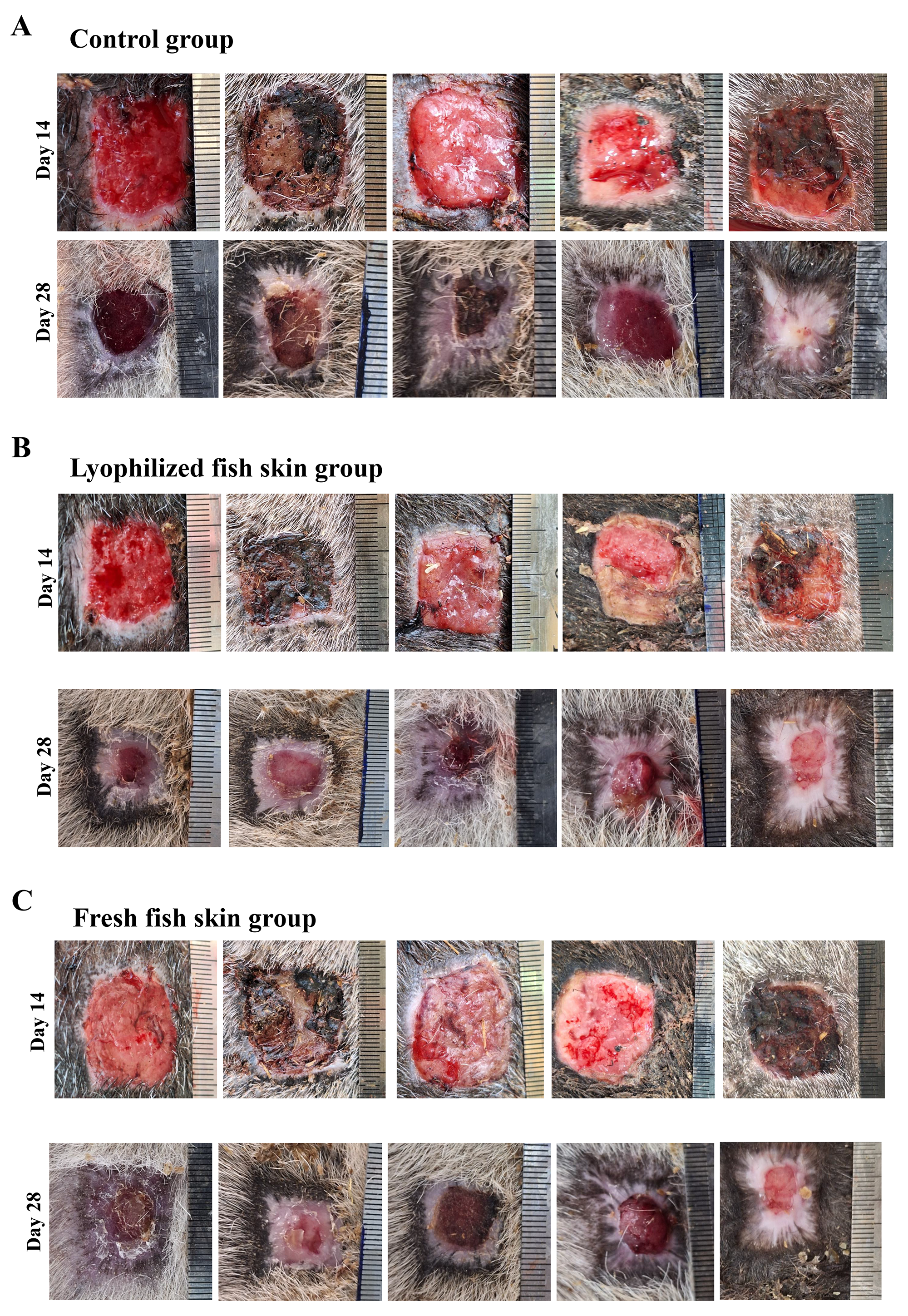

Supplement: Supplementary file 1 — (PNG 853 KB) [file 11259_2025_10821_Fig6_ESM.png]

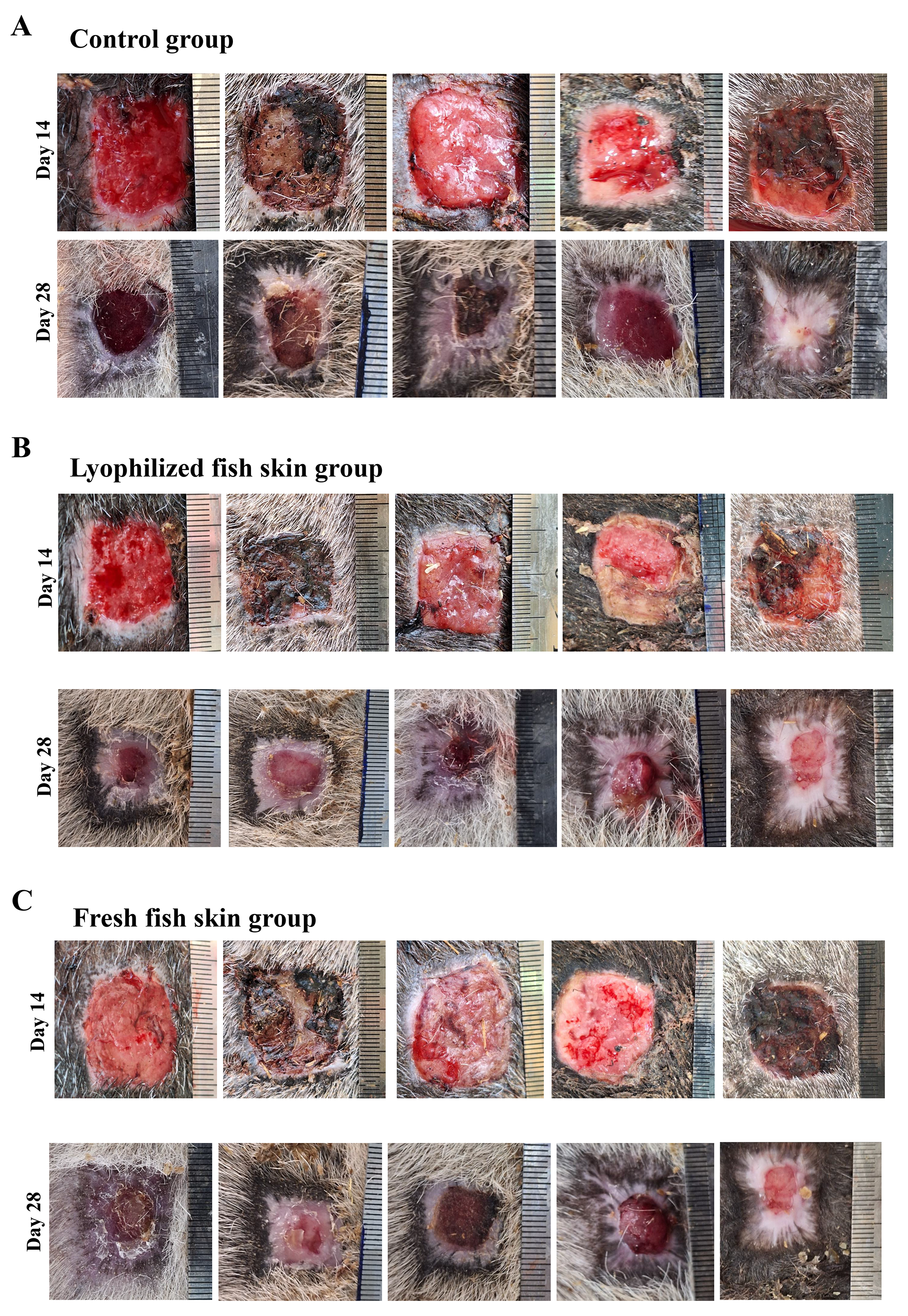

Supplement: Supplementary file 2 — High Resolution Image (26.4 MB) [file 11259_2025_10821_MOESM1_ESM.tif]
